# A Novel Levothyroxine Solution Results in Similar Bioavailability Whether Taken 30 or Just 15 Minutes Before a High-Fat High-Calorie Meal

**DOI:** 10.1089/thy.2021.0604

**Published:** 2022-08-09

**Authors:** Murray Ducharme, Claudia Scarsi, Elisa Bettazzi, Giuseppe Mautone, Yvette Lewis, Francesco S. Celi

**Affiliations:** ^1^ Faculté de Pharmacie, University of Montreal and Learn and Confirm, Inc., St. Laurent, Quebec, Canada.; ^2^ IBSA Institut Biochimique SA, Lugano, Switzerland.; ^3^ Learn and Confirm, Inc., St. Laurent, Quebec, Canada.; ^4^ Division of Endocrinology Diabetes and Metabolism, Department of Internal Medicine, Virginia Commonwealth University, Richmond, Virginia, USA.

**Keywords:** food effect, hypothyroidism, levothyroxine, pharmacokinetics, solution

## Abstract

**Background:**

Levothyroxine (LT4) sodium is a standard treatment for hypothyroidism. Its absorption and bioavailability when taken as a tablet have been shown to be significantly decreased with concomitant food ingestion. Therefore, LT4 formulations are recommended to be taken on an empty stomach, at least 30, ideally 60, minutes before breakfast, potentially affecting adherence to therapy. A novel LT4 solution (Tirosint^®^-SOL) has been shown to result in a faster absorption process than tablets or soft-gel capsule formulations. The objective of this trial was to evaluate the bioavailability of this preparation taken 15 minutes before a high-fat high-calorie meal in comparison with the minimally recommended 30-minute interval.

**Methods:**

Thirty-six (33 completers, 24 males and 9 females) healthy volunteers participating in the randomized study took 600 mcg of LT4 oral solution, single doses after a 10-hour fast, 15 or 30 minutes before a high-fat, high-calorie, FDA-approved standardized meal in a controlled research setting. We measured serum total thyroxine using Liquid Chromatography with Tandem Mass Spectrometry at baseline and multiple time points up to 72 hours after LT4 administration. The predefined equivalence boundaries for the extent of exposure reflected by the area under the curve (AUC) were 80–125%. The washout period was at least 35 days.

**Results:**

The geometric mean ratios and confidence intervals (CIs) for the baseline-adjusted extent of exposure represented by AUCs truncated at both 48 and 72 hours after dosing (AUC_0–48_: 90% [90% CI 86–94]; and AUC_0–72_: 92% [90% CI 87–97]) were within the prespecified equivalence boundaries. The baseline-adjusted peak concentration was also clinically similar (C_max_: 85% [90% CI 80–90]). The median t_max_ was 1.5 hours in each group. The rate of adverse events was similar between groups.

**Conclusions:**

We observed no significant difference in the pharmacokinetic properties of a novel LT4 solution administered 15 and 30 minutes before a high-fat high-calorie meal in normal subjects. Further research is needed to evaluate (a) the differences in overall bioavailability at other time points (including immediately premeal) and (b) the real-world effectiveness of this preparation in newly proposed administration conditions to optimize treatment outcomes in hypothyroid patients.

## Introduction

The thyroid hormones, triiodothyronine (T3) and thyroxine (T4), are pivotal to activation of metabolic processes and overall well-being (1). T3, representing ∼20% of the hormones secreted by the thyroid gland, is mainly derived from metabolism of T4 by deiodination (1). Treatment of hypothyroidism can therefore rely on the sole administration of levothyroxine (LT4).

While untreated hypothyroidism can be fatal (2), recent studies have demonstrated that even mild hypothyroidism or hyperthyroidism in undertreated or overtreated patients may be associated with adverse health outcomes and excess mortality (3,4).

LT4 sodium is the standard of care for treatment of hypothyroidism and is available in various forms, including tablets, soft-gel capsules, and solutions (2). LT4 formulations are made of synthetic LT4 and, being chemically equivalent, exert the same physiological effect as endogenous T4, thus maintaining crucial hormone balance (5).

LT4 bioavailability is significantly decreased with food (6). Coadministration of LT4 tablets with a high-fat high-calorie meal significantly reduced T4 C_max_ by 40–49% and AUC_0–48_ by 38–40% with respect to fasting conditions (7). For this reason, LT4, in any dosage form, is recommended to be administered in the fasting state at least 30, ideally 60, minutes before breakfast (8).

Indeed, the American Thyroid Association guidelines for treatment of hypothyroidism recommend that LT4 be consistently taken either 60 minutes before breakfast or at bedtime (≥3 hours postdinner) for optimal consistent absorption (9). These strict recommendations are inconvenient for patients, who may find them challenging to work around their daily schedule, to the point where 20% or more do not comply with the dosing recommendations (10). Nonadherence to LT4 therapy is quite common (11,12), potentially contributing to difficulties in achieving and maintaining therapeutic targets.

For the purpose of reducing certain inconveniences for patients and improving their adherence to treatment, other conditions of LT4 administration have been studied, such as concomitantly with breakfast (13,14), in the evening (15–17), or even as a single weekly administration (18,19).

A new oral solution of LT4 (manufactured by IBSA, Institut Biochimique SA) has been recently approved and commercialized under the name Tirosint^®^-SOL (including Tirosintsol, Tirosint Solution, Syntroxine Sol, Levotirsol, Synotirex, Tirosol, Solsint, and Tsoludose). This solution has been shown to reach maximum systemic exposure 30 minutes earlier than LT4 tablets and soft-gel capsules and has a 30-minute shorter lag time (20–22).

Quicker absorption of the solution may therefore allow administration closer to breakfast time as absorption may start immediately once LT4 reaches the gut, upon gastric emptying, and before coming into contact with food. This may be more convenient for patients and may favor their adherence to treatment.

The objective of this study was to investigate the impact of administering the LT4 oral solution 15 minutes before a standardized meal compared with the minimum recommendation of 30 minutes in the current FDA-approved insert.

## Materials and Methods

### Study design

Healthy male and female volunteers, aged 18–50 years, took part in a phase I, single-center, randomized, open-label, single-dose, two-period, two-sequence, crossover pharmacokinetic (PK) study to compare the PK profile of the LT4 solution administered as a single 600-mcg oral dose at 15 and 30 minutes before consuming a standard, FDA-approved, high-fat high-calorie meal intended to mimic the worst-case scenario of breakfast (23–26).

### Ethics

All clinical work was conducted in compliance with good clinical practices, as referenced in the International Council for Harmonisation guidelines (ICH E6), with principles of Good Laboratory Practices, and the Declaration of Helsinki. The study was reviewed by the Advarra Institutional Review Board in Ontario, Canada (Approval No. Pro00042379). Informed consent was obtained from all subjects before study enrollment.

### Population

Subjects were healthy volunteers with a body–mass index between 18.5 and 30.0 kg/m^2^, weighing >50 kg for males and >45 kg for females, and were non- to moderate smokers (≤9 cigarettes/day).

Subjects were not eligible for enrollment in the study if they had significant disease (particularly cardiac disease) or any clinically significant abnormality or met any of the additional exclusion criteria listed in the protocol.

### Study restrictions

Subjects were prohibited from eating foods known to affect absorption of LT4 and the PKs of drugs in general. Smoking (maximum nine cigarettes/day) was not permitted within two hours before dosing until four hours postdose.

### Treatments

A single 600-mcg dose of the LT4 solution (4 unit-dose ampules of 150 mcg/mL) was administered after a 10-hour fast, either 15 minutes (Treatment A) or 30 minutes (Treatment B) before a high-fat high-calorie meal, as required in food effect studies. The meal consisted of 150 calories derived from proteins, 250 calories from carbohydrates, and 500–600 calories from fat, totaling about 800–1000 calories (two fried eggs, two strips of bacon, two slices of buttered toast, hash browns 120 g, and whole milk 200 mL), and had to be consumed within 30 minutes (24–27).

### Study procedures

The screening visit occurred within 28 days before first dose administration. All subjects were admitted to the clinic at least 10 hours before drug administration and remained onsite until the 48-hour postdose blood draw for each period, reflecting a highly controlled research setting. The 72-hour postdose samples were collected on return visits. Serum samples were taken at three different times at baseline, and at 0.5, 1, 1.5, 2.5, 3, 4, 6, 8, 10, 12, 16, 24, 48, and 72 hours after dosing. The washout period was at least 35 days.

The study lasted ∼2.5 months in total. Serum concentrations of total (bound and free) T4 were measured using a validated Liquid Chromatography with Tandem Mass Spectrometry method. Safety monitoring included adverse event (AE) monitoring, clinical laboratory results (i.e., hematology, biochemistry, urinalysis, and serology), vital signs, electrocardiogram, and physical examinations. Treatment-emergent AEs (TEAEs) were defined as AEs that occurred on or after study drug administration and were classified according to the MedDRA^®^ dictionary, version 23.0.

### PK parameters

PK parameters were calculated using standard noncompartmental methods for total T4, using serum concentrations and baseline-adjusted serum concentrations. For baseline correction, the baseline value (mean of 3 predose samples) was subtracted from each measured concentration for each subject and in each period.

Parameters included maximum observed concentration (C_max_), time of observed maximum concentration (t_max_), and area under the concentration–time curve from time 0 to 48 hours (AUC_0–48_) and to 72 hours (AUC_0–72_).

### Statistical methods

Demographic parameters, TEAEs, and PK parameters were summarized descriptively for all dosed subjects. Statistics included arithmetic and geometric means, standard deviation (SD), coefficient of variation (CV), and minimum, median, and maximum values.

AUC_0–48_, AUC_0–72_, and C_max_ were compared between treatment groups using the general linear model ANOVA procedure in SAS^®^, with an alpha error of 0.05. The model included sequence, period, treatment, and subject*sequence as fixed effects. Intrasubject CV was estimated from the ANOVA residual error. The ratios of geometric means (Treatment A/Treatment B) and their corresponding 90% confidence intervals [90% CIs] were calculated.

Lack of clinical difference between treatment groups was *a priori* set to be declared if the 90% CIs calculated for the area under the curves (AUCs) were contained within the predefined standard limits of 80–125%. Wilcoxon's test was performed on t_max_.

## Results

### Population

**FIG. 1.**
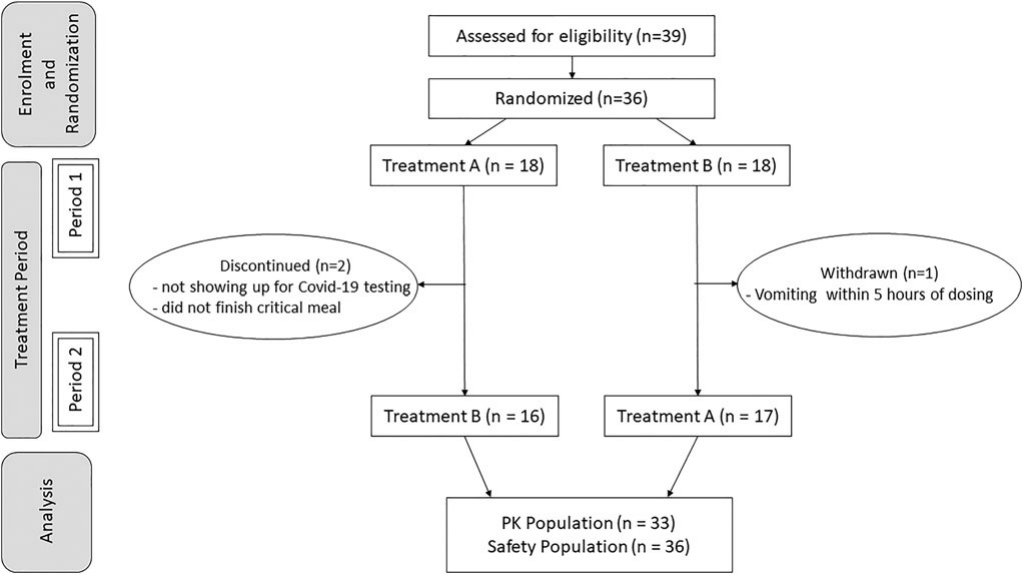
Flow diagram for the comparative bioavailability study of levothyroxine sodium oral solution administered 15 and 30 minutes before a meal in fasted state. *n* = number of subjects, PKs, pharmacokinetics; Treatment A, levothyroxine administered 15 minutes before the meal; Treatment B, levothyroxine administered 30 minutes before the meal.

Table 1 summarizes the demographics of the study participants. A total of 36 healthy male and female subjects were dosed in the study. Three subjects did not complete the trial because of absence at COVID-19 testing, vomiting <5 hours after dosing, and incomplete consumption of the critical meal, seen in Figure 1, and were excluded from the PK analysis as per protocol. Additionally, one subject missed the 72-hour blood sampling during one study period.

**Table 1.** Summary of Demographic and Body Measurement Data of Subjects Included in the Pharmacokinetic Population

Parameter	Category	PK population* N* = 33
Age, years	Mean ± SD	38 ± 8
	Range	23–49
	Median	39
Gender^a^	Female	9 (27.3)
	Male	24 (72.7)
Race^a^	Asian	1 (3.0)
	Black/African American	2 (6.1)
	White	29 (87.9)
	Other	1 (3.0)
Ethnicity^a^	Not Hispanic or Latino	32 (97.0)
	Hispanic or Latino	1 (3.0)
BMI, kg/m^2^	Mean ± SD	25.73 ± 2.76
	Range	19.08–29.73
	Median	25.96
Height, cm	Mean ± SD	170.8 ± 8.7
	Range	153.0–189.5
	Median	170.0
Weight, kg	Mean ± SD	75.33 ± 11.68
	Range	53.40–98.40
	Median	75.50
TSH, mIU/L	Mean ± SD	1.88 ± 0.92
	Range	0.65–4.15
	Median	1.55
Thyroxine, free, pmol/L	Mean ± SD	14.64 ± 1.47
	Range	12.00–17.60
	Median	14.60
Triiodothyronine, total, nmol/L	Mean ± SD	1.66 ± 0.34
	Range	1.10–2.36
	Median	1.59

^a^ N(%).

BMI, body–mass index; *N*, number of observations; SD, standard deviation; TSH, thyrotropin.

The PK population included 33 healthy participants who completed both periods and had an adequately characterized PK profile. The safety analysis included 36 participants who had received at least one dose of study medication.

### Pharmacokinetics

**FIG. 2.**
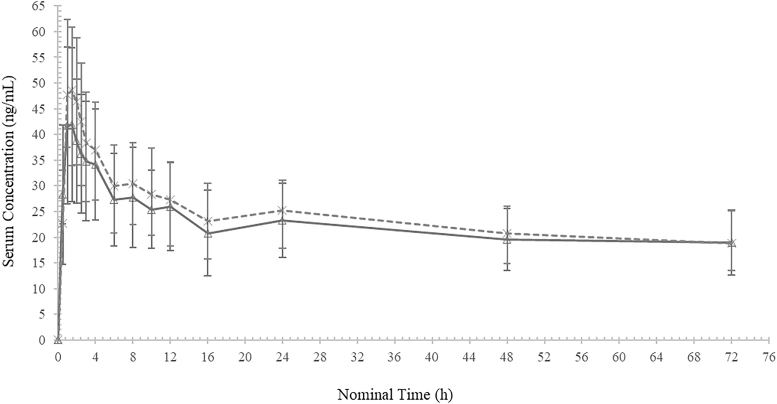
Mean ± SD total levothyroxine (baseline adjusted) concentrations—linear scale. 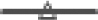 Levothyroxine sodium administered 15 minutes before breakfast (A). 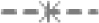 Levothyroxine sodium administered 30 minutes before breakfast (B). SD, standard deviation.

The mean serum total T4 concentration–time profiles (baseline adjusted) for both treatments are presented in Figure 2. The profiles appeared similar, whether the LT4 solution was administered 15 or 30 minutes before the meal.

Descriptive statistics for total T4 PK parameters (baseline adjusted) are presented in Table 2. The arithmetic mean AUC_0–72_ was 1623 h × ng/mL with a CV% of 27.1 for Treatment A (15 minutes before the meal) and 1725 h × ng/mL with a CV% of 21.8 for Treatment B (30 minutes before the meal).

**Table 2.** Descriptive Statistical Summary of Total Levothyroxine Pharmacokinetic Parameters (Baseline Adjusted)

Parameter, unit	Treatment									
	A (15 minutes)					B (30 minutes)				
	*N*	Arithmetic		Geometric		*N*	Arithmetic		Geometric	
		Mean	CV%	Mean	CV%		Mean	CV%	Mean	CV%
AUC_0–48_, h × ng/mL	33	1141.19	29.22	1093.48	30.77	33	1239.97	22.25	1210.13	22.89
AUC_0–72_, h × ng/mL	32	1622.86	27.14	1565.75	27.81	33	1724.83	21.83	1684.38	22.64
C_max_, ng/mL	33	44.80	32.30	42.50	34.74	33	51.80	26.09	50.00	28.15
t_max_,^a^ hours	33	1.486 (0.985, 7.980)				33	1.486 (0.985, 5.987)			

^a^ Median (min, max) presented for t_max_.

Treatment (A): levothyroxine sodium 4 mL × 150 mcg/mL oral solution, 15 minutes before breakfast.

Treatment (B): levothyroxine sodium 4 mL × 150 mcg/mL oral solution, 30 minutes before breakfast.

AUC, area under the curve; CV, coefficient of variation; Max, maximum; Min, minimum; *N*, number of observations.

The least square mean ratios (Treatment A/Treatment B) and 90% CIs from the ANOVA are provided in Table 3. The geometric mean ratios for AUC_0–48_ and AUC_0–72_ were determined to be ∼90% and 92%, respectively. The 90% CIs for AUC_0–48_ and AUC_0–72_ were entirely contained within the standard equivalence interval of 80–125%. The intrasubject variability was 11.19% for AUC_0–48_ and 13.60% for AUC_0–72_.

**Table 3.** Summary of Geometric Mean Ratios (Treatment A/Treatment B) and 90% Confidence Intervals for Comparison of Levothyroxine Administration at 15 and 30 Minutes (Baseline-Adjusted Data)

Parameter, unit	Geometric LSM		GMR,*^a^*%	90% CI^b^		Intrasubject CV, %
	Treatment A, 15 minutes	Treatment B, 30 minutes		Lower, %	Upper, %	
AUC_0–48_, h × ng/mL	1087.85	1209.44	89.95	85.85	94.24	11.19
AUC_0–72_, h × ng/mL	1551.95	1693.93	91.62	86.50	97.04	13.60
C_max_, ng/mL	42.29	49.97	84.64	79.98	89.58	13.64

^a^ Geometric mean ratio.

^b^ Ninety percent confidence interval for the GMR.

Treatment (A): levothyroxine sodium 4 mL × 150 mcg/mL oral solution, 15 minutes before breakfast.

Treatment (B): levothyroxine sodium 4 mL × 150 mcg/mL oral solution, 30 minutes before breakfast.

LSM, least squares mean.

Results indicate that the overall extent of exposure can be considered clinically similar whether the solution was taken 15 or 30 minutes before the meal.

The geometric mean ratio for C_max_ was determined to be ∼85%, while the median t_max_ was similar between the two treatments at 1.5 hours (*p* > 0.05).

### Safety

Table 4 summarizes the AEs of all subjects who received at least one dose of study medication. A total of 11 TEAEs were reported (9 of 36 participants). In the Treatment A group, there were a total of three TEAEs (one case had increased blood pressure and two cases had headache) that were possibly related to the treatment. In the Treatment B group, there were a total of three TEAEs (one case had increased body temperature, one had vomiting, and one had intermittent headache) that were possibly related to the treatment. There were no serious AEs for either treatment.

**Table 4.** Summary of Possibly Related Adverse Events by Treatment

System organ class/MedDRA^®^ preferred term	Treatment group	
	A, 15 minutes	B, 30 minutes
Number of subjects dosed	35	34
Gastrointestinal disorders		
Vomiting	0	1 (2.9%)
Investigations		
Blood pressure increased	1 (2.9%)	0
Body temperature increased	0	1 (2.9%)
Nervous system disorders		
Headache	2 (5.7%)	1 (2.9%)
SAEs	0	0

Treatment (A): levothyroxine sodium 4 mL × 150 mcg/mL oral solution, 15 minutes before breakfast.

Treatment (B): levothyroxine sodium 4 mL × 150 mcg/mL oral solution, 30 minutes before breakfast.

MedDRA^®^, Medical Dictionary for Regulatory Activities, version 23.0; SAEs, significant adverse events.

Overall, these findings suggest that administration of LT4 in a single 600-mcg dose is safe and well tolerated in healthy subjects.

## Discussion

LT4 is a lifelong medication that has to be taken daily (8). Nonadherence rates reported by clinicians range from 22% to 82%, and as such, many strategies have been tested to improve adherence (28). LT4 is recommended to be taken on an empty stomach since its absorption is known to be greatly impacted by food (6–8). Studies have shown that a 30- to 60-minute time interval between drug administration and food intake may be difficult for patients to observe, often leading to noncompliance with this strict recommendation (10,12).

In a study by El Helou *et al.*, the majority of patients (98.5%) took LT4 in the morning (91.4% before meals), with 51.1% patients taking LT4 close to food intake. The overall percentage of low adherence to LT4 therapy was found to be at least 54.9%, and it was concluded that adherence to LT4 treatment could be improved by educating patients about treatment requirements (e.g., drug–food interactions) and by introducing new treatment regimens (12).

McMillan *et al.* surveyed 925 hypothyroid patients and found that 20% took LT4 with their meals, while another 21.5% ingested their LT4 dose <30 minutes before a meal. Not surprisingly, 13.4% of patients experienced difficulties controlling their hypothyroid symptoms (10). Additionally, it is commonly recognized that poor adherence is one of the main causal factors of inadequate thyroid hormone replacement (8,28). As observed in other therapeutic areas, one crucial factor affecting medication adherence is the timing of drug administration (29,30).

Topaloğlu *et al.* evaluated adherence to treatment in pregnant women with primary hypothyroidism. Of 85 women, 42.4% had low adherence to LT4 and 41.17% had out-of-range thyrotropin (TSH) levels. Although no association was found between adherence and treatment success, it is interesting to note that a reduced interval between ingestion of LT4 and breakfast was associated with improved adherence (medium/high) to LT4 (31).

Recent studies have suggested that the effect of food on LT4 could be minimized by replacing tablets with an oral solution (32). When administered as an oral solution, LT4 is absorbed more quickly than in tablet form (33) and is less affected by gastric pH (34) and other factors affecting its gastrointestinal absorption (35–37). The LT4 solution was compared with LT4 tablets in patients and it appeared to be more effective in lowering TSH levels in malabsorption and nonmalabsorption patients alike (37–39).

In another study, hypothyroid patients were asked if it was difficult to take LT4 tablets minimally 30 minutes before breakfast or coffee. Most patients found it challenging and preferred the possibility of taking the LT4 solution with breakfast (40).

As it can be difficult for patients to strictly respect the conditions surrounding coadministration of LT4 and food, this study investigated whether a novel LT4 liquid formulation could be administered as early as 15 minutes before a high-fat high-calorie meal versus the recommended minimum wait of 30 minutes. The 15-minute time interval was selected as the midpoint between the currently recommended minimum interval of 30 minutes for this particular LT4 formulation and concomitant meal administration, allowing for at least partial gastric emptying after LT4 administration and before food intake.

In our assessment, it was considered a reasonable improvement to a patient's tight morning routine, with the intention of improving patient adherence, although data on subject preferences regarding the timing of administration of the preparation were not collected. In view of the extremely high volume and calorie density of the FDA-approved meal composition for food interaction studies, administering LT4 with the meal was not considered for this study.

Furthermore, the meal administered in the study may not necessarily reflect a typical meal of LT4-treated patients in real life. As absorption was observed to be equivalent at 15 and 30 minutes, we postulate that the therapeutic target may be more easily maintained in those patients who have difficulty waiting before breakfast.

To our knowledge, this was the first PK study to examine the effect on the bioavailability and absorption of LT4 when reducing the time interval between administration of LT4 and consumption of a high-fat high-calorie meal, conducted with an adequate number of subjects and under highly standardized conditions. The design of the study was appropriate given its purpose and in agreement with bioequivalence guidance (23–25).

The composition, calories, and fat content of the meal are not typical for breakfast; however, they are specifically recommended by the FDA and EMA to represent the worst-case scenario in food effect studies. The physiological conditions induced by a high-fat meal generally provide the greatest effects on gastrointestinal physiology and the maximum effects on the systemic availability of drugs (26).

The investigation in healthy volunteers rather than hypothyroid patients ensured that there were no medical conditions or concomitant medications that could significantly impact LT4 PKs and bias the results of this study, allowing for high standardization. Moreover, PK studies in healthy volunteers are the recommended methodology to evaluate the effect of food on drug absorption (26).

The study design administered a high single dose of 600 mcg in healthy volunteers since their individual serum concentration–time profiles for PK assessment have to be baseline adjusted to account for endogenous T4 to accurately detect the orally administered LT4 and ensure that the comparative relative bioavailability results are robust (23). The washout period was at least 35 days between treatments due to the long elimination half-life of T4 (6–9 days) and to ensure that a significant period effect would not be seen (23).

The overall extent of exposure in the present study was investigated using a truncated AUC up to 72 hours because differences between formulations of the same active ingredient (here LT4) can only result in differences in the absorption process, not in the elimination, and would therefore have been detectable within the first 72 hours after dosing.

It should be noted that C_max_ and t_max_ were not considered to be clinically relevant with respect to the assessment of PK and therapeutic equivalence, as LT4 is used chronically and is a prohormone of T3. C_max_ is mainly related to the safety of LT4; therefore, presumably a lower C_max_ could be potentially beneficial rather than detrimental. In addition, the peak generated by exogenous administration of LT4 does not mimic the physiological conditions, whereas normal endogenous T4 levels remain quite stable over the day (41).

In some patients, LT4 can be administered once or twice a week as a large dose rather than smaller daily doses (18,19,42,43), suggesting that the effect is related more to the total amount absorbed and its bioavailability than to peak concentrations reached.

There are several limitations of this study. One limitation was not assessing results under total fasting conditions (i.e., continued fasting four hours postadministration). This extreme condition is quite uncommon in real life, therefore it was considered to be decidedly less relevant clinical information for physicians and patients alike. Furthermore, other relevant time points, such as one hour before breakfast or taking the medication with breakfast, were not evaluated.

External generalizability of our findings to real-world hypothyroid patients is also limited, given that our study was conducted in healthy subjects under highly controlled study conditions. Generalizability may be particularly limited to hypothyroid patients with comorbidities who may be on multiple concurrent medications and whose diets may vary from that examined in the study.

Another limitation is that the assumption of improved convenience and potentially improved compliance with a 15-minute time frame for LT4 administration before breakfast was based on investigators' opinions and not formally studied in LT4-treated hypothyroid patients.

In conclusion, in this randomized, controlled, PK crossover study, the baseline-adjusted systemic exposure profiles of a novel LT4 solution were considered equivalent when administered to healthy subjects 15 or 30 minutes before a high-fat high-calorie meal in a highly controlled research setting. We thus propose that the interval between administration of this LT4 solution and food intake may be shortened from 30 to 15 minutes.

We believe that reducing this time interval could potentially improve convenience for patients and possibly facilitate adherence to therapy. Further research is needed to evaluate the differences in overall bioavailability at other time points, including immediately before breakfast.

Furthermore, research is needed to evaluate the real-world effectiveness of this novel LT4 preparation in the newly proposed administration conditions to optimize treatment outcomes in hypothyroid patients.
